# Co-Created Messaging for Influenza Vaccination in a High-Risk Hispanic Community Provides Groundwork for COVID-19 Vaccine

**DOI:** 10.1089/heq.2020.0132

**Published:** 2021-05-24

**Authors:** Apple Long, Sheryl Mathew, Kristin S. Alvarez, Jillian Smartt, Monal Shah, Christopher Madden, Trish M. Perl, Fred P. Cerise, Kavita P. Bhavan

**Affiliations:** ^1^ Department of Internal Medicine, University of Texas Southwestern Medical Center, Dallas, Texas, USA.; ^2^ Center for Innovation and Value, Parkland Health and Hospital System, Dallas, Texas, USA.; ^3^ Health Systems Operations Administration, UT Southwestern Medical Center, Dallas, Texas, USA.

**Keywords:** influenza vaccination, health disparities, community-based health care innovations, social marketing in health care

## Abstract

**Purpose:** Influenza/pneumonia is the eighth leading cause of death in the United States. The 2020–2021 influenza season is predicted to be further impacted by COVID-19 infections. Historical data reflect disproportionate morbidity and mortality rates in the Hispanic population for influenza and COVID-19. Influenza vaccination rates remain low in the Hispanic community. We aim to improve vaccination through a community-led event, partnering with the Cristo Rey School Dallas, located in a zip code with a higher age-adjusted influenza/pneumonia mortality rate. A survey was administered to adults attending the Influenza vaccine event to understand attitudes and perceptions about influenza, vaccination, and effective messaging strategies for the campaign.

**Methods:** Messaging was cocreated with student health ambassadors to promote immunization and delivered through trusted sources. The health department administered vaccines to individuals >age 3 at no cost. Adults were asked to complete a 19-question survey postvaccination offered in both English and Spanish.

**Results:** Two hundred and forty-one of 394 (61.2%) participants completed the survey. Ninety-eight percent identified as Hispanic/Latino, and the majority of surveys were administered in Spanish. Among Spanish language participants, the church bulletins (57.3%) and Spanish language radio (30.5%) were reported to be most effective modes of messaging versus word of mouth (32.9%) and social media (26.3%) for English-speaking participants. Sixteen percent of participants surveyed had never received an influenza vaccine before this event.

**Conclusion:** Cocreated messaging delivered by trusted sources in the Hispanic community led to a successful Influenza vaccine drive with the Dallas County health department.

## Introduction

There were more than 117,000 deaths reported from influenza in the United States over the last three Influenza seasons.^1,2^ While vaccination can prevent cases, hospitalizations, and deaths due to influenza,^3^ <50% of the U.S. population receives the vaccine. Racial disparities exist for Influenza vaccination across the country, with lower rates reported among Black and Hispanic populations.^4–6^ The Centers for Disease Control (CDC) fluvax report highlights a persistent trend over the last decade (2009–2018). The most recent influenza season (2018–2019) demonstrated the highest rate of influenza vaccination in non-Hispanic White persons (53%), 41% in non-Hispanic Black persons, and 38% in Hispanic persons. Influenza season data from 2018 to 2019 show higher rates of hospitalization due to influenza for non-Hispanic Black (68%) and Hispanic populations (44%) compared with the non-Hispanic White population (38%).^7^ Common barriers to vaccine uptake include poverty, lack of health insurance, access to care, low levels of educational attainment, language differences, and misunderstanding vaccination risks.^8^

With the emergence of the COVID-19 global pandemic, mortality from respiratory infections has risen dramatically, with more than 340,000 deaths in the United States attributed to the COVID-19 virus. The 2020–2021 influenza season is challenged by the presence of COVID-19 with a greater burden of respiratory infections posing a threat to health care resources and increased morbidity and mortality.^9^ In this context, it is increasingly important to promote influenza vaccine uptake in racial and ethnic minority communities with known health disparities and vaccine hesitancy.

Parkland Health and Hospital System (PHHS) in conjunction with the Dallas County Health and Human Services (DCHHS) department publishes a Community Health Needs Assessment for Dallas County every 3 years to assess the current state of health, identify health disparities, and implement strategies to address community needs. In the most recent report from 2019, 41 zip codes were recognized as having a high SocioNeeds Index, calculated from six indicators: poverty, income, unemployment, occupation, education, and language.^10^ The 75217 zip code in southeast Dallas has multiple health disparities, including the fourth lowest life expectancy and an observed influenza and pneumonia age-adjusted mortality rate 2.3 times higher than other areas in Dallas County.^10^

Bilingual messaging and education have been shown to improve influenza vaccine confidence and uptake across the U.S. Hispanic population and locally in studies of Texas communities.^11–16^ Padilla and colleagues highlight another barrier in their study associating an individual's decision not to vaccinate with trust in the government. The authors recommend improved therapeutic relationships between health care providers and patients as a means to address such mistrust and build confidence in vaccine safety and efficacy.^13^ The importance of this therapeutic relationship is further supported by Mark and Paramore's study where the absence of a regular health care provider was found to be a significant barrier to vaccination.^12^

Alternatively, cultural factors such as religious faith may be positively associated with vaccine uptake. Among Mexican-born Hispanic immigrants, for example, regular church attendance was associated with higher rates of influenza vaccination, suggesting that targeting church attendees would also be beneficial.^12^

Building on prior research, we developed a community-based culturally specific messaging and delivery strategy to improve vaccination rates in this underserved region. A postimmunization survey was developed and administered to gain a better understanding of knowledge, attitudes, and perceptions of the influenza vaccine among participants at this community-driven event.

## Methods

### Setting

Through an existing partnership with the Cristo Rey Dallas Preparatory School located within the 75217 zip code, DCHHS in partnership with PHHS planned to administer 400 no-cost influenza vaccines at a community event hosted at the high school in January 2020. The influenza vaccine drive was held on a Sunday before and after church services near the school to improve participation. While the health department planned four vaccination events in the 75217 zip code over the last influenza season, our community-based collaborative approach was only taken for one pilot event. A survey assessing knowledge, attitudes, and perceptions about the influenza vaccine was developed in English and Spanish and approved by the institutional IRB for program evaluation, incorporated into Research Electronic Data Capture (REDCap), and administered to participating adults after they were immunized.

### Messaging

**FIG. 1.**
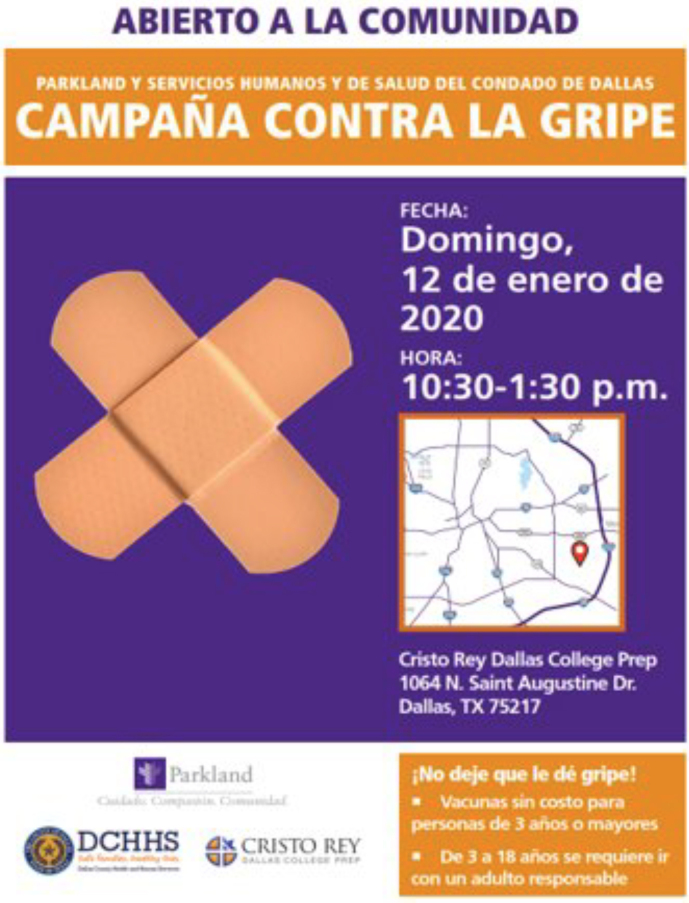
Example of a nonthreatening church bulletin to promote the Influenza vaccine drive. This nonthreatening flyer was utilized in Facebook, Instagram, and LinkedIn campaigns to promote consistent advertising.

Student members of Cristo Rey High School's Medical Society club were invited to participate in this project based on their interest in health care careers. Recruited students fluent in both English and Spanish were asked to serve as health ambassadors, a role created for this pilot program. Health ambassadors were educated about the influenza virus, morbidity and mortality associated with infection, and the importance of vaccination as a preventive measure. Messaging was then cocreated by health ambassadors in English and Spanish for print and digital media outlets. This information was subsequently disseminated through trusted community sources, including the local Catholic Church, Spanish language radio, food banks and grocery stores, and the Mexican Consulate. Spanish radio station advertisements were delivered by student health ambassadors and flyers were created with positive images (with the intent to reduce fear, i.e., bandage vs. syringe, Fig. 1) and posted at school, church bulletins, and other aforementioned trusted sources. Uniform messaging was also disseminated through social media outlets, including Facebook, Instagram, Twitter, and LinkedIn. Information related to the influenza vaccination event was provided through multiple sources over a 3-week period.

### Survey

A postimmunization 19-question survey asking questions on knowledge, attitudes, and perceptions about the influenza vaccine was administered to participants ≥18 years of age, utilizing the REDCap (https://www.project-redcap.org/) database hosted at PHHS.^17^ Health ambassadors administered the survey, interviewing participants in their preferred language (Spanish or English). The questionnaire comprised items measuring the source of information about the drive, prior year influenza shot history, recipients' perceptions of influenza vaccine effectiveness, and barriers to acquiring the immunization. Select demographics were also collected from respondents, including age range, gender, marital status, ethnicity, employment, education level, and insurance status.

### Statistical analysis

R (list version, https://www.r-project.org/) was utilized for statistical analysis.^18^ Descriptive analyses included means for continuous variables and percentages for categorical data. To test for differences among survey question responses, Fisher's exact test was used as appropriate. For assessing the relationships between questionnaire responses and also between questionnaire responses and actual measures, each answer choice was analyzed independently using Fisher's exact test. Student's *t*-test was utilized for survey responses that were scaled (i.e., 1–5 scale) with a parametric distribution. One-sided *p*-values of <0.05 were considered to be statistically significant.

## Results

Of the four no-cost influenza vaccination events administered by DCHHS in the 75217 zip code for the 2019–2020 influenza season, the health ambassador-led event resulted in the highest turnout, with 394 participants vaccinated over 4 h. The other three events utilized a standard health system (DCHSS)-led messaging approach, resulting in 300 cumulative participants and only 27 participants vaccinated in the event immediately before the community-engaged influenza drive at Cristo Rey High School. A total of 36 volunteers and staff were present at the high school to assist DCHHS personnel with Influenza vaccine administration, with half of this group comprising students who also helped promote the event. Print media was disseminated through weekly church bulletins and school newsletters in addition to a local food bank over a 3-week period. Two high school students recorded a 30-sec radio advertisement, which aired 18 times in a week on Spanish Contemporary and 45 times in a week over Spanish Contemporary and Mexican Regional Radio. Social media messages were developed by a group of high school students and posted 2 days before the event, resulting in 1132 Instagram impressions with a reach of 964 unique users and 8451 Facebook impressions with a reach of 8282 unique users.

Of the 394 participants attending the event, 274 individuals (70%) were subsequently found to have an existing medical record in our electronic medical record, indicating care delivery (ranging from a single emergency department visit to patients with an established primary care provider at PHHS). Following vaccination, adults were invited to participate in a survey at the event and 241 (61.2%) individuals completed the survey. Among surveyed participants, 98% identified as Hispanic or Latino and 68% requested that the survey be administered in Spanish. The majority of respondents were married (72.2%) and female (59.8%), between 30 and 64 years of age (Table 1). While data were skewed toward women, subsequent analysis of responses to questions by gender showed no significant difference for all but one question. Specifically, women stated that the influenza vaccine was more effective in response to the question: “How effective do you think the Influenza shot is at preventing the Influenza on a scale of 1–5?” (mean effective scale of 4.1 vs. 3.8, on a scale of 1–5, with 1 being the least effective and 5 being extremely effective, *p* = 0.012). Educational attainment at the level of 8th–12th grade was reported by nearly half of those surveyed (47.7%). A smaller proportion of participants reported having a salaried job (43.2%), and 15% reported being self-employed (Table 1). Reasons for a high unemployment rate in this sample could not be determined in the absence of questions related to this issue. The survey was designed to provide a snapshot of individuals attending the pilot event and maximize participation. Consistent with population census data, only 10% of participants reported a form of health insurance (either Medicare/Medicaid or commercial/private, 4.1% and 5.4% respectively). The reasons for low government insurance rates, while unknown, may be a reflection of Texas not having expanded Medicaid and perhaps the immigration status of participants. Nine percent of participants stated “Other” for health insurance, which may designate charity funding provided by Parkland based on eligibility (Table 1). It is important to note, however, the Parkland Financial Assistance designation is not equivalent to insured status. This finding is also consistent with zip code census data, suggesting that over-representation of uninsured people among those surveyed is unlikely. The majority of survey participants reported hearing about the influenza drive through community messaging by advertisements on Spanish language radio 61%) and/or flyers on local church bulletins (48.1%).

**Table 1.** Demographics and Characteristics of 241 Influenza Vaccination Participants

Characteristics	*n* (%)
Gender	
Female	144 (59.8)
Male	97 (40.2)
Survey language	
Spanish	165 (68.5)
English	76 (31.5)
Ethnicity	
Hispanic/Latino	236 (97.9)
Non-Hispanic White	3 (1.3)
Non-Hispanic Black or African American	1 (0.4)
Asian/Pacific Islander	1 (0.4)
Age bracket, years	
18–29	27 (11.2)
30–44	99 (41.1)
45–64	98 (40.7)
65+	17 (7.1)
Marital status	
Married	174 (72.2)
Single	49 (20.3)
Separated/divorced	12 (5.0)
Widowed	6 (2.5)
Work status	
Salary	104 (43.2)
Unemployed	89 (36.9)
Self-employed	36 (14.9)
Cannot work	12 (5.0)
Education	
Up to 8th grade	115 (47.7)
Up to high school/GED	99 (41.1)
Up to college	13 (11.2)
Insurance	
Medicare/Medicaid	10 (4.1)
Commercial/private	13 (5.4)
Uninsured	196 (81.3)
Other	22 (9.1)
Prior influenza vaccine ever	203 (84.2)
Prior influenza vaccine in the past year	90 (37.3)

GED, General Education Development.

A comparison of responses between participants completing the survey in English versus Spanish showed no significant difference in patterns of vaccine uptake. While 84% of all respondents reported receiving the influenza vaccine at some time in their life, only 37% received the vaccine in the prior year (Table 1), with no statistical difference between Spanish and English responders. Similarly, the predominant response for groups was yes when asked if they were likely to get a Influenza vaccine the following year (98.2% and 94.7% in Spanish and English survey responders, respectively) (Table 2).

**Table 2.** Influenza Vaccine Perceptions Between Spanish and English Survey Participants

Survey question	Spanish survey		English survey		*p*
	*n*	Value (%)	*n*	Value (%)	
^a^How did you hear about the Influenza drive?	164		76		
Church bulletin		94 (57.3)		22 (28.9)	<0.001
Spanish radio advertisement		50 (30.5)		11 (14.4)	0.010
Family/friend (word of mouth)		20 (12.2)		25 (32.9)	<0.001
Social media (Facebook/Instagram)		18 (11)		20 (26.3)	0.004
Cristo Rey student		11 (6.7)		8 (10.5)	0.31
Community flyer		4 (1.2)		4 (2.4)	0.27
E-mail (Mexican Consulate)/letter home		1 (0.6)		4 (2.4)	0.04
Have you ever had the Influenza shot?—*n* (%)	165		76		0.26
Yes		142 (86)		61 (80.3)	
No		23 (14)		15 (19.7)	
^a^If no, why?—*n* (%)	23		15		
I could not afford it		2 (8.7)		3 (20)	0.36
No health insurance		3 (13)		0 (0)	0.26
Work hours prevented me from getting it		3 (13)		3 (20)	0.66
Childcare		0 (0)		0 (0)	1.0
I did not know I needed it		9 (39.1)		11 (73.3)	0.05
Afraid of getting sick		3 (13)		2 (13.3)	1.0
I did not think it would work		6 (26.1)		4 (26.7)	1.0
I did not have transportation		1 (4.3)		0 (0)	<0.001
^a^Why did you get the Influenza shot today?—*n* (%)	163		76		
The Influenza vaccine is free		56 (34.4)		44 (57.9)	<0.001
Day/time was convenient		40 (24.5)		44 (57.9)	<0.001
Location was convenient		42 (25.8)		42 (55.3)	<0.001
A health care professional told me to		28 (17.2)		6 (7.9)	0.07
Other		63 (38.7)		6 (7.9)	<0.001
How effective do you think the Influenza shot is at preventing the Influenza on a scale of 1–5?—mean (±SD) 1—not effective, 5—extremely effective.	165	4.15 (±0.71)	76	3.6 (±0.91)	<0.001
How sick do you think the Influenza would make you feel if you did not get the Influenza shot?—mean (±SD) 1—not sick, 5—extremely sick.	165	4.0 (±1.0)	76	3.1 (±1.19)	<0.001
Do you have adults aged 65+ years and/or small children living in your home?—*n* (%)	165		76		0.78
Yes		90 (54.5)		43 (56.6)	
No		75 (45.5)		33 (43.4)	
If you have adults aged 65+ years or small children at home, how sick do you think they would get if they got the Influenza from you?—mean (±SD) 1—not sick, 5—extremely sick.	90	4.1 (±0.9)	43	2.7 (±1.2)	<0.001
Will you get your Influenza vaccine next year?—*n* (%)	165		76		0.21
Yes		162 (98.2)		72 (94.7)	
Not sure		3 (1.8)		4 (5.3)	
No		0 (0)		0 (0)	

^a^ Allowed to choose multiple responses. % of total individuals.

SD, standard deviation.

**FIG. 2.**
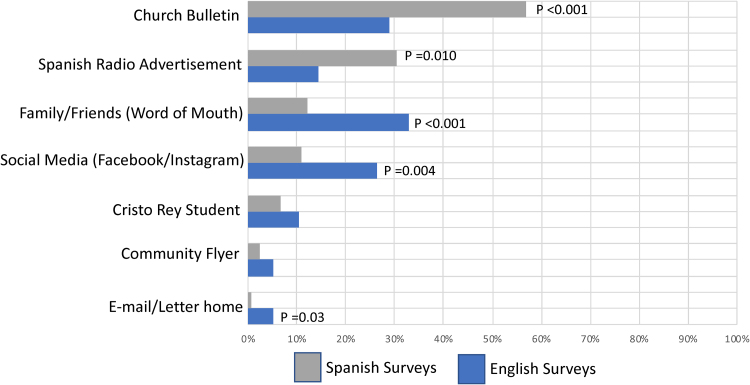
How did survey participants hear about the Influenza vaccine drive? *Gray and blue bars* represent response percentages of the total number of participants with either English or Spanish surveys. Participants were allowed to choose more than one answer choice for this question. The Fisher exact test was used to calculate *p*-values in comparing responses between English and Spanish surveys.

Spanish survey and English survey respondents were found to differ on questions related to messaging sources (Fig. 2) and vaccine efficacy. Spanish respondents were more likely to have heard about the vaccination event through the church bulletin (57.3%), followed by Spanish radio (30.5%) (*p*≤0.001 and 0.010, respectively). English language responders, on the other hand, were more likely to have heard about the event through social media sites and family/friends (*p*=0.004 and <0.001, respectively) (Table 2). The two groups also differed in response to perceived effectiveness of the influenza vaccine. Spanish language responders stated that the vaccine was more effective in preventing disease (mean effective scale of 4.15 vs. 3.6, on a scale of 1–5, with 1 being the least effective and 5 being extremely effective, *p*<0.001) (Table 2). Consistently, Spanish language responders were more likely to state that they would be sicker if they did not receive the vaccine compared with English responders (mean effective scale of 4.0 vs. 3.1, on a scale from 1 to 5, 1 being not sick and 5 being extremely sick, *p*<0.001) (Table 2). Of the total population surveyed, 55.2% lived with either small children or adults over 65 years of age. In this subset, Spanish language participants were more likely than English language participants to report concern for infecting a family member if they were to contract influenza (mean effective scale of 4.1 vs. 2.7, on a scale from 1 to 5, 1 being not sick and 5 being extremely sick, *p*<0.001) (Table 2).

## Discussion

Messaging and delivery with community engagement were vital components to the success of our influenza vaccine drive. Promoting health and wellness with low- and high-tech modalities and outlets was effective for this population (Fig. 1). Educating high school students about the importance of vaccination represents an opportunity to transfer knowledge and build capacity from within the community toward a culture of better health. Spanish language radio advertising prompted enthusiastic participation from high school students, their families, and the larger community and appeared to be more effective than routine campaigns by health systems and public health departments. Location and timing also contributed to the success of a large vaccination turnout. Following church services on Sunday, participants were able to walk over to the adjacent school and receive their Influenza vaccine with relative ease.^13^

Our findings from survey participants are consistent with prior research showing that Mexican-born immigrants have greater confidence in vaccine safety compared with individuals from the community born in the United States.^11^ Cohesive social networks may play a role in driving health-protective sentiments as regular attendance in church is also associated with or trended toward increased vaccination rates.^11,13^ This protective effect may be diluted with the acculturation of immigrants,^19^ perhaps through influences of social media,^22,24^ as demonstrated by attitudes of the English language survey cohort (Table 2).

An interesting finding from the survey was a greater likelihood among English versus Spanish language participants to receive messaging through social media. Although social media has become a vital tool to promote health care initiatives,^11,20,21^ there is an increasing rate of misinformation generated on social media platforms,^22^ related to vaccine efficacy and safety,^23–25^ and the fidelity of information on the internet is often not validated. For users with nonmedical education, it is difficult to judge the reliability of health information from these sources, creating an environment ripe for conspiracy theories, disinformation, and honest misinformation.

Strengths of this assessment include the use of cocreated, culturally sensitive, and language-specific messaging with community engagement to promote influenza immunization in a largely Hispanic community known to have increased morbidity and mortality from influenza and pneumonia. While previous studies have looked at promoting influenza vaccination within racial and ethnic communities with health disparities,^26–28^ there remains much to be learned about knowledge, perceptions, and attitudes toward vaccination among uninsured Hispanic populations. Our postimmunization survey was designed to ask these questions at a single point in time among adults participating in this vaccination event. A major limitation to the study is applicability of survey data due to a self-selected population of participants. The high percentage of individuals who reported having previously received an influenza vaccine indicates that those with prior vaccine exposure may have been more likely to participate in the vaccine drive and are over-represented in our sample. Yet, interestingly, the percentage of individuals who received the influenza vaccine in the past year (37.3%) is on par with the national average of Hispanic adults (37.1%).^7^ Since our survey only queried individuals who had already received the vaccine that day, our data may additionally over-represent positive sentiments toward the influenza vaccine as compared with the generalized community. Last, only 7.1% of participants were age 65+, suggesting the need for improved targeted messaging in this population.

Similar to influenza seasons of the past, racial and ethnic minorities again bear a disproportionate burden of illness and death from COVID. Data collected through the CDC's COVID-NET registry updated November 30, 2020, reflect these disparities, with Hispanic or Latino persons being 1.7 times likely to have a diagnosis, 4.1 times more likely to be hospitalized, and 2.8 times more likely to die from COVID-19 compared with White non-Hispanic persons.^29^ The dual impact of COVID-19 and influenza for the 2020–2021 season thus requires optimization of all preventive measures, including influenza vaccine uptake in vulnerable communities.

Although the COVID-19 vaccine is new, it is not immune to misinformation and will require effective messaging for successful uptake.^30,31^ Disparities are again described for intent to be vaccinated against COVID-19, with a cross-sectional survey by Fisher et al. identifying race and the absence of influenza vaccination in the prior year to be independently associated with vaccine hesitancy.^32^ In another recent study comparing reported past influenza vaccination with acceptance of a COVID-19 vaccine, unemployed participants reported lower influenza vaccine uptake and lower COVID-19 vaccine acceptance compared with employed and retired individuals.^33^ We describe a similar population with a high rate of unemployment among adults surveyed at the vaccination event and many individuals reporting not being immunized against influenza in the past year. Our community-based approach for the influenza vaccine may then provide a relevant framework for COVID-19 vaccine uptake in this population. The church, for example, is a culturally relevant source of trust and was instrumental to the success of our messaging strategy. Prior studies have demonstrated the effectiveness of church-based interventions among Hispanic/Latino populations for chronic disease and health promotion measures.^34,35^

Effective community engagement is essential to reshape culturally relevant messaging to promote immunization against infection. History has shown that getting the science right in vaccine development is simply not enough if we are to promote successful uptake among vulnerable populations. For example, while the influenza vaccine at no cost represents an opportunity to achieve *health equity*, we continue to see poor uptake among Hispanic communities known to have increased morbidity and mortality. Health care systems and providers can begin to bridge an existing gap, recognizing the importance of both the *message* and *messenger* when designing campaigns to increase trust, confidence, and ultimately improved vaccination rates for communities with health disparities.

## Abbreviations Used

CDC: Centers for Disease Control
DCHHS: Dallas County Health and Human Services
GED: General Education Development
PHHS: Parkland Health and Hospital System
REDCap: Research Electronic Data Capture
SD: standard deviation
